# Voxki: A Text-to-speech Anki Add-on with Built-in Medical Pronunciation Support

**DOI:** 10.1007/s40670-026-02890-6

**Published:** 2026-09-21

**Authors:** Tobias Whitford, Aidan Pech, Christian Donlon, Chase Goldberg, Etienne Mill

**Affiliations:** New York Medical College, School of Medicine, Valhalla, NY 10595 USA

**Keywords:** Spaced repetition, Text-to-speech, Medical education, Accessibility, Educational technology

## Abstract

Voxki is a free, offline Anki add-on providing automatic text-to-speech for medical flashcards, with 396 curated medical pronunciation corrections, context-aware acronym handling, and zero-configuration setup. Developed and refined through anonymous user feedback, it supports auditory access to card content for medical students, including those with visual impairments.

**Supplementary Information:**

The online version contains supplementary material available at https://doi.org/10.1007/s40670-026-02890-6.

## Introduction

Spaced repetition has emerged as one of the most effective strategies for long-term retention of dense material in medical education [1]. Anki, an open-source spaced repetition flashcard program, has been widely adopted; a 2022 single-institution study found that approximately 70% of first-year medical students used Anki by their 13th week of medical school [2], and Anki use has separately been associated with improved academic performance in medical school [3]. Two main card styles used by Anki users include basic, which is a typical flashcard format with information on the front and back of the cards and cloze deletion, which is a card with blanks for the user to fill in before revealing the answer. Despite widespread use, the platform has notable accessibility gaps. Students with visual impairments, those unable to view their devices during study sessions, and learners who rely on or benefit from auditory reinforcement lack adequate text-to-speech (TTS) support, despite evidence that text-to-speech and related read-aloud tools can improve reading comprehension for students with reading disabilities [4]. Existing TTS add-ons such as AwesomeTTS and HyperTTS are designed for foreign language learning rather than specialized domains like medicine: they require cloud-based API keys, cost money for premium voices, necessitate editing card templates or pre-generating audio files, and frequently mispronounce medical terminology, including eponyms, abbreviations, and Latin-derived terms, which may confuse learners, particularly those early in training.

**Table:** Comparison of Voxki with existing general-purpose Anki TTS add-ons

Dimension	AwesomeTTS / HyperTTS	Voxki
Cost	Free core, but premium cloud voices incur per-character or subscription costs	Free, no premium tier
API requirements	Cloud-based API key required for most higher-quality voices	None — uses the operating system’s native TTS engine
Offline use	Limited; higher-quality voices depend on an internet connection	Fully offline
Cloze handling	Generic playback; not aware of hint/blank conventions	Cloze-aware: reads hints or substitutes “blank,” then reads the revealed answer
Medical pronunciation support	None; general-purpose voices frequently mispronounce medical terms	396 curated pronunciation corrections
Privacy	Card content and audio may be sent to third-party cloud APIs	Fully on-device; no card content, audio, or usage data leaves the device
Setup burden	Moderate–high; API key configuration, card-template editing, or pre-generated audio required	Zero-configuration; works immediately after installation
Customization	Voice and provider selection	Speech rate, per-feature toggles, editable pronunciation dictionary, configurable hotkeys
Mispronunciation reporting	Not built in	In-app mispronunciation reporting form

AwesomeTTS/HyperTTS characteristics reflect their general-purpose design as described in their public documentation; verify each cell against current AwesomeTTS/HyperTTS documentation before submission, as add-on capabilities may change

To address this gap, we developed Voxki, a free, open-source Anki add-on that provides text-to-speech functionality designed for medical students. Voxki requires no configuration. Upon installation via the standard AnkiWeb add-on process, the program automatically reads card content aloud during review. For cloze-deletion cards, the most common format in medical Anki decks, Voxki reads the hint aloud when available or substitutes the word “blank” when no hint is present on the question side, then reads the revealed answer on the answer side. Standard flashcards are read in their entirety on both sides. The add-on automatically detects and skips image-occlusion cards, which are visual and not suited for audio playback.

## The Innovation

**Fig. 1:**
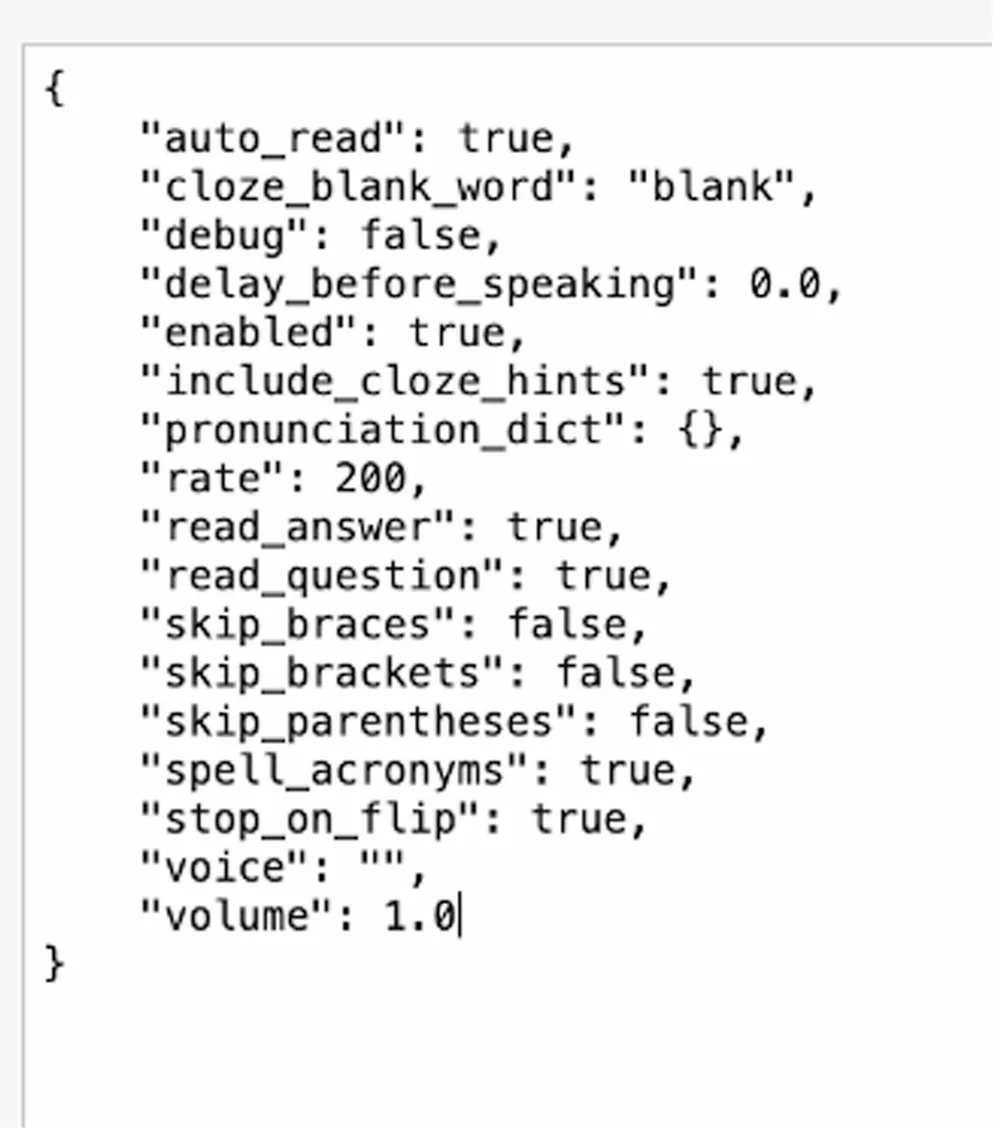
The Voxki settings panel within the Anki desktop application, showing speech-rate presets, voice selection, and the pronunciation-dictionary editor

The built-in dictionary contains 396 curated pronunciation corrections (Table 1), added only where the native engine audibly misreads a term, spanning abbreviations, eponyms, organisms, and mnemonics; entries are expanded, letter-spelled, or respelled per term, and users can edit or extend the dictionary, with user entries taking precedence. Beyond static dictionary lookups, Voxki employs context-aware acronym processing: when encountering an uppercase term, the program analyzes surrounding words to determine whether it is a true acronym that should be spelled out letter by letter or a stress marker in a phonetic pronunciation guide that should be left intact. The add-on also detects mnemonic acronym patterns commonly used in medical education (such as MUDPILES or SIGECAPS) and adjusts its handling of single-letter cloze deletions accordingly. Users can customize their experience through a built-in settings panel (Fig. 1) that offers adjustable speech rate with preset options, configurable answer-reading modes, including a full-sentence option for cloze cards, and a pronunciation dictionary editor for adding custom terms. A mispronunciation reporting form is accessible directly from the add-on’s menu to facilitate ongoing improvement. Voxki operates entirely offline using the operating system’s native TTS engine (macOS say, Windows SAPI, Linux eSpeak) — streamed for near-instant onset on macOS and Linux and file-based on Windows — with no account, internet access, or cost; no card content, audio, or usage data leaves the device. It is distributed and updated through AnkiWeb, and full technical specifications appear in Supplementary Table S1, prepared in alignment with the TIDieR [5], GREET [6], and STARE-HI [7] reporting guidelines.

From a theoretical standpoint, Voxki leverages the modality effect as described in Baddeley and Hitch’s Working Memory Model. By pairing visual text with simultaneous auditory input, the add-on engages both the visuospatial sketchpad and the phonological loop, distributing cognitive processing across two working memory subsystems rather than relying on a single channel. This parallel engagement is theorized to strengthen encoding and reduce cognitive load during the review of dense material and is the rationale behind the creation of Voxki [8]. This benefit is not automatic: audio that is poorly timed, redundant with on-screen text, or otherwise distracting can increase extraneous cognitive load rather than reduce it [9, 10]. Accordingly, the working-memory framework is presented here as a design rationale rather than evidence of an established learning benefit, pending formal evaluation of learning outcomes.

## Implementation

Compatibility with existing medical school decks was a core design priority. Voxki was developed against the AnKing Step deck, the most widely used deck among medical students preparing for USMLE Step examinations, and functions with any user-created or community deck without modification. Beyond its utility for auditory learners, Voxki addresses an important accessibility need for students with visual impairments or reading disabilities, supporting auditory access to card content and reduced visual demand during review, though the Anki interface itself remains visually operated. Non-native English speakers may also benefit from hearing unfamiliar medical terms spoken aloud. Since public release on AnkiWeb in January 2026, feedback has been collected through three channels: an anonymous, voluntary online form (61 responses), public AnkiWeb reviews (34 ratings, 29 positive, as of August 2026), and community discussion. Pronunciation reports formed the largest category of form responses and drove iterative dictionary corrections. Public reviews described faster review sessions and studying while walking or fatigued, although no learning outcome has been formally measured. A June 2026 feature release introduced regressions — re-reading of question text and vocalization of formatting artifacts — which users reported within days; the previous stable release was restored while those features are re-engineered. The pronunciation dictionary has additionally been adopted by an independently developed mobile companion add-on. Limitations include calibration of overrides against the macOS engine, no support for regional pronunciation variants (rendering depends on the user’s selected system voice), the absence of formal pronunciation validation, unsupported progressive-reveal card types, no screen-reader compatibility testing beyond the operating system’s native screen reader, no in-review pause/replay control beyond Anki’s native review controls, no formally evaluated alternative pathway for students who cannot use audio-only review beyond disabling the add-on and reviewing cards visually as before [11], no controlled comparison, potential OS-dependent voice-quality differences across the macOS, Windows, and Linux native engines, the possibility of novelty or selection bias among users who volunteered feedback, and the absence of outcome data. Formal evaluation of learning outcomes is a planned next step and should assess usability, acceptability, accessibility, pronunciation accuracy, cognitive load, card completion rate, study frequency, delayed recall, and comparison to Anki without Voxki or to other TTS tools [12]. Voxki is freely available on AnkiWeb, and the source code is openly accessible.

**Table 1:** Sample curated pronunciation corrections in Voxki (v1.2.3)

Medical term	Voxki rendering	Behavior	Category
CABG	cabbage	R	Abbreviation
HFpEF	H F pef	L + R	Abbreviation
HFrEF	H F ref	L + R	Abbreviation
Sjögren	show grins	R	Eponym
Guillain-Barré	ghee yan bah ray	R	Eponym
Behçet	beh chets	R	Eponym
C. diff	see diff	R	Organism
H. pylori	H pie lore ee	L + R	Organism
HbA1c	hemoglobin A 1 C	E + L	Laboratory value
ACEi	ace inhibitor	E	Abbreviation
SLE	S L E	L	Abbreviation

Renderings are reproduced verbatim from the v1.2.3 dictionary. *E *expanded to the full term; *L *letter-spelled; *R *respelled phonetically; combinations occur. Overrides were added when the developer heard the native macOS engine misread a term during his own card review, with systematic completion of affected notation families, or when users reported a misreading through a voluntary, anonymous feedback form; reports were reviewed and incorporated where confirmed. Respelling targets follow General American conventions calibrated against the macOS engine; final rendering depends on the user’s selected system voice, and regional variants are not separately supported. Users can edit and extend the dictionary, and user entries take precedence. No formal validation against a reference pronunciation source has been performed

## Conclusion

Voxki is a promising, free, offline, open-source Anki add-on designed to improve medical pronunciation support and auditory access for medical students. By integrating a specialized, offline text-to-speech engine with over 400 built-in medical pronunciation mappings and smart cloze-deletion handling, the add-on could be used to bridge the gap between spaced repetition software and auditory learning modalities. This free, open-source tool may serve as a vital resource for medical students managing dense curricula, auditory learners, individuals with visual or reading impairments, and non-native English speakers seeking standardized pronunciation. Limitations of the current study are inherent in innovation pieces, in that there is no formal outcome data or controlled comparison in the effect of the add-on. Also, we have yet to formally test accessibility and externally validate the pronunciation of terms, including regional variations. Future directions include continuous dictionary expansion and refinement driven by user metrics and community-sourced mispronunciation reporting. Formal evaluation of usability, accessibility, pronunciation accuracy, learner experience, and learning outcomes is needed before any claims of improved study efficiency or retention can be made.

## Supplementary Information

Below is the link to the electronic supplementary material.

Supplementary Material 1

## Data Availability

Voxki is freely available on AnkiWeb (add-on 1810680328).
