# Supplementary material for: Voxki: A Text-to-speech Anki Add-on with Built-in Medical Pronunciation Support
Source: Med Sci Educ. 2026 Sep 21;36(6):2979–82. doi: 10.1007/s40670-026-02890-6 (PMC13759952; doi:10.1007/s40670-026-02890-6)
Supplement: Supplementary file 1 [file 40670_2026_2890_MOESM1_ESM.docx]

# **Supplementary Table S1**

**Supplementary Table S1. Implementation summary for Voxki, aligned with TIDieR, GREET, and STARE-HI reporting domains**

| **Domain** | **Description** |
| --- | --- |
| Purpose | On-demand audio narration of flashcards during review, supporting pronunciation exposure and reduced visual demand. No claims of improved retention are made. |
| Intended users | Medical students using cloze-based decks (e.g., AnKing); compatible with any Anki deck. |
| Setup | Single-step installation from AnkiWeb (add-on 1810680328); functional with default settings; no account required. |
| Technical requirements | Anki [range per current AnkiWeb listing] on macOS, Windows, or Linux; native operating-system TTS engines (macOS say, Windows SAPI, Linux eSpeak); no internet connection required after installation. |
| Privacy | All speech synthesis occurs on-device; no card content, audio, or usage data is transmitted; no telemetry is collected. Donation and feedback links open only on explicit user action. |
| Pronunciation dictionary | 396 versioned entries; selection and review as described in the Table 1 note; user-defined entries take precedence and persist across updates. |
| Cloze behavior | Question side: cloze deletions voiced as a configurable word (default "blank"), with optional hint reading; single-letter hints announced as "starts with…"; answer side reads revealed content. Image-occlusion cards are detected and skipped. |
| Customization | Speech rate with presets, voice selection, per-feature toggles, editable pronunciation dictionary, configurable hotkeys. |
| Accessibility | Supports auditory access to card content; not a screen-reader substitute — Anki's interface remains visually operated. |
| Planned evaluation | [e.g., structured usability evaluation; pronunciation-accuracy audit against a reference source; user-reported outcomes] |
